# Transcriptome analysis of human monocytic cells infected with *Burkholderia* species and exploration of pentraxin-3 as part of the innate immune response against the organisms

**DOI:** 10.1186/s12920-019-0575-7

**Published:** 2019-09-06

**Authors:** Sophie A. Aschenbroich, Eric R. Lafontaine, Maria Cecilia Lopez, Henry V. Baker, Robert J. Hogan

**Affiliations:** 1 Department of Pathobiological Sciences, School of Veterinary Medicine, University of Wisconsin-Madison, 2015 Linden Drive, Madison, WI 53706 USA; 2 Department of Infectious Diseases, College of Veterinary Medicine, University of Georgia, 501 D.W. Brooks Drive, Athens, GA 30602 USA; 3 Department of Molecular Genetics and Microbiology, College of Medicine, University of Florida, 2015 SW 16th Ave, Gainesville, FL 32608 USA; 4 Department of Veterinary Biosciences and Diagnostic Imaging, College of Veterinary Medicine, University of Georgia, 501 D.W. Brooks Drive, Athens, GA 30602 USA

**Keywords:** *Burkholderia mallei*, *Burkholderia thailandensis*, Transcriptome, Human monocytes, PTX3, Pattern recognition receptor, Opsonophagocytosis, Intracellular survival

## Abstract

**Background:**

*Burkholderia mallei (Bm)* is a facultative intracellular bacterial pathogen causing highly-fatal glanders in solipeds and humans. The ability of *Bm* to thrive intracellularly is thought to be related to exploitation of host immune response-related genes and pathways. Relatively little is known of the molecular strategies employed by this pathogen to modulate these pathways and evade intracellular killing. This manuscript seeks to fill gaps in the understanding of the interface between *Bm* and innate immunity by examining gene expression changes during infection of host monocytes.

**Methods:**

The transcriptome of *Bm*-infected human Mono Mac-6 (MM6) monocytes was profiled on Affymetrix Human Transcriptome GeneChips 2.0. Gene expression changes in *Bm*-infected monocytes were compared to those of *Burkholderia thailandensis (Bt)*-infected monocytes and to uninfected monocytes. The resulting dataset was normalized using Robust Multichip Average and subjected to statistical analyses employing a univariate F test with a random variance model. Differentially expressed genes significant at *p <* 0.001 were subjected to leave-one-out cross-validation studies and 1st and 3rd nearest neighbor prediction model. Significant probe sets were used to populate human pathways in Ingenuity Pathway Analysis, with statistical significance determined by Fisher’s exact test or z-score.

**Results:**

The Pattern Recognition Receptor (PRR) pathway was represented among significantly enriched immune response-related human canonical pathways, with evidence of upregulation across both infections. Among members of this pathway, pentraxin-3 was significantly upregulated by *Bm*- or *Bt*-infected monocytes. Pentraxin-3 (PTX3) was demonstrated to bind to both *Bt* and *Burkholderia pseudomallei (Bp)*, but not *Bm*. Subsequent assays did not identify a role for PTX3 in potentiating complement-mediated lysis of *Bt* or in enhancing phagocytosis or replication of *Bt* in human monocytes.

**Conclusion:**

We report on the novel binding of PTX3 to *Bt* and *Bp*, with lack of interaction with *Bm*, suggesting that a possible evasive mechanism by *Bm* warrants further exploration. We determined that (1) PTX3 may not play a role in activating the lytic pathway of complement in different bacterial species and that (2) the opsonophagocytic properties of PTX3 should be investigated in different primary or immortalized cell lines representing host phagocytes, given lack of binding of PTX3 to MM6 monocytes.

## Background

*Burkholderia mallei* (*Bm*) is a gram-negative, encapsulated, facultative intracellular bacillus and host-adapted deletion clone of the environmental pathogenic organism, *Burkholderia pseudomallei* (*Bp*) [1–3]. *Bm* is responsible for causing glanders, a highly contagious and fatal zoonotic disease affecting solipeds and humans, against which no effective vaccine exists [2]. Transmission of glanders to susceptible hosts occurs through ingestion, aerosol, or percutaneous routes, and disease manifestations are characterized by respiratory, cutaneous, and lymphatic, ulcerative lesions, abscesses, or granulomas, and/or septicemia [2, 4]. Glanders can manifest as an acute (mules and donkeys) or chronic (horses) disease, with nearly 90% of horses developing chronic or latent infections prior to death and, thus, serving as reservoirs for the maintenance and spread of the disease [2, 4]. *Bm* can chronically persist within host tissues following apparent clinical resolution [2]. The ability of *Bm* to thrive within host cells and tissues is thought to be related to evasion or exploitation of immune response-related signaling pathways. Altogether, a more thorough understanding of the *Bm*-host interface is urgently needed as part of the effort to develop novel medical countermeasures against this agent [5].

Relatively little is known regarding *Bm-*induced immunomodulation of host genes and pathways that allows for evasion of innate immunity, successful intracellular survival, and persistence within host tissues [6, 7]. To date, the molecular interface between *Bm* and host immune cells has been predominantly examined from a bacterial genetics standpoint and largely within murine systems [8–16], with a primary focus on characterizing the role of *Bm* virulence factors in mechanisms of host cell adherence and invasion, as well as intracellular survival [8–16]. However, the potential mechanisms by which *Bm* may modulate host genes and pathways to promote intracellular survival still remain poorly defined. Topological analyses of *Bm*-host protein interactions performed to fill this knowledge gap have provided evidence for *Bm* proteins to target intracellular host immune response signaling processes, with possible interactions identified between TRAF-6 and IκBα and the *Bm* protein, BMAA0728 (TssN) [17, 18]. Together with these in silico analyses, other studies aimed at better defining the interface between *Bm* and innate immunity have primary focused on the molecular impact of *Bm* intracellular infection on cellular activation and/or select cytokine profiles in vitro. These studies demonstrated that *Bm*-infected macrophages produce reduced levels of both IFN-dependent genes and mediators as well as cytokines and expressed iNOS only at higher multiplicities of infection and later time points after intracellular infection, as compared to *Escherichia coli*-infected murine macrophages [7]. Furthermore, *Bm*-infected primary human monocyte-derived macrophages and alveolar type II pneumocytes released high levels of IL-10 in the early stages of infection, with a delayed induction of pro-inflammatory cytokines (IL-6, TNF-α) [6]. This altered cytokine signaling is thought to aid *Bm* in successful intracellular replication prior to host detection and the development of an effective immune response [6]. The work presented herein seeks to extend on the studies by Brett et al. 2008 and Lu et al. 2012 by investigating the potential for modulation of host immune response-related genes and pathways during intracellular survival, by assessing global host transcriptional changes during intracellular infection of monocytes on a genome-wide scale, in the biologically-relevant human host. In particular, this study focuses on characterizing bacterial modulation of host innate immunity both at the pathway and gene-level.

The long pentraxin-3 (PTX3) is a critical component of innate immunity against microorganisms and a soluble pattern recognition receptor (PRR) rapidly produced by diverse cell types including myeloid cells, endothelial cells, epithelia, and fibroblasts [19, 20]. Pentraxin-3 production by these cells is enhanced by pro-inflammatory stimuli or by direct recognition of microbes or microbial components, leading to significant increases in plasma from physiological concentrations (~ 2 ng/mL) to 200–800 ng/mL [19, 20]. Following its production, PTX3 actively participates in microbial recognition and opsonization, complement activation and modulation, opsonophagocytosis, and host resistance to select pathogens in vivo [21–26]. Specifically, PTX3 has the capacity to recognize and bind to several bacterial, fungal, and viral agents, namely *Neisseria meningitidis*, *Aspergillus fumigatus*, and influenza virus, or to microbial moieties, such as *Klebsiella pneumoniae* outer membrane protein A [20, 22–29] and synergize with the host complement system to enhance deposition of complement initiators (mannose-binding lectin, Ficolin-2) and central or downstream complement effectors (C3, C4) onto microbial surfaces, such as *Aspergillus fumigatus* and *Candida albicans* [30, 31]. Beyond the recruitment of complement to the surface of microorganisms, the capacity for PTX3 to additionally coordinate the terminal lytic complement pathway has been briefly examined in the context of *Pseudomonas aeruginosa* [23]. Cytolytic studies with normal human serum did not demonstrate a role for this PRR in mediating amplification of the lytic phase of complement against this bacterium [23]. Apart from interactions with complement, PTX3 also exhibits opsonic properties capable of enhancing the phagocytosis of several microbial agents, such as *Pseudomonas aeruginosa* RP73, *uropathogenic Escherichia coli* CFT073, and *Aspergillus fumigatus,* by host neutrophils and macrophages [22, 23, 25–27, 31], a property that has been suggested to be dependent on active complement and accessible FcγRs [23, 26]. Beyond its ability to potentiate opsonophagocytosis, some studies demonstrate a role for PTX3 in modulating phagocyte activation and antimicrobial defenses to enhance intracellular killing of opsonized and phagocytosed microorganisms [22, 27]. Finally, PTX3 has also been described to have systemic effects as an immunotherapeutic in multiple rodent models of infection, and its therapeutic activity in vivo is thought to be based on its ability to promote a balanced Th1-mediated inflammatory response and modulate the recruitment, phagocytosis, and antimicrobial activity of inflammatory cells [21–26]. Altogether, while PTX3 has been studied in the context of several microbial agents, the interactions between PTX3 and pathogenic *Burkholderia* sp., such as *Bm* or *Bp*, or the non-pathogenic, related organism, *Burkholderia thailandensis* (*Bt)*, have not yet been investigated. Therefore, this research presents novel findings related to the interface between PTX3 and *Bt*, *Bm*, or *Bp* and the potential relevance of these interactions to host innate immune response defense mechanisms.

## Methods

### Cell culture

The human Mono Mac-6 (MM6) monocytic cell line was used to study the interaction of *Bm* with human monocytes. MM6 monocytes were also infected with the genetically-related, non-pathogenic *Bt*, as a comparison. Mono Mac-6 cells were obtained from the German Culture Collection (DSMZ no.: ACC 124), Braunschweig, Germany and were maintained in 24-well plates in 1 mL of RPMI 1640 containing 25 mM HEPES, supplemented with 10% fetal calf serum, 1% L-Glutamine, 1% non-essential amino acids, 1% sodium pyruvate, and 10 μg/mL of recombinant human insulin (Santa Cruz Biotechnology, CAS 11061–68-0) in a humidified 5% CO_2_ atmosphere at 37 °C. Cell densities were maintained between 2.5 × 10^5^–1.0 × 10^6^ cells/mL, passaged every 3–4 days, and cultured until approximately passage 35. *Bm* ATCC23344 was cultured at 37 °C using Brucella medium (BD) supplemented with 5% (vol/vol) glycerol. *Bt* DW503, an *amrAB-oprA* efflux pump mutant of the parental strain *Bt* E264 that is highly sensitive to kanamycin, was cultured at 37 °C using Luria-broth (LB) medium. *Bp* K96243 was included in the binding assays and was prepared from cultures grown on Trypticase Soy Agar (BD) at 37 °C.

### Intracellular survival assays

These assays were developed to study host monocyte immune response gene or pathway regulation by *Bm* in comparison to *Bt*, to gain potential insights into pathogenic mechanisms specific to *Bm* or common to both *Burkholderia* species. MM6 monocytes were seeded in a 24-well plate at 1 × 10^6^ cells/mL prior to infection with *Bt* DW503 or *Bm* ATCC23344. Bacterial suspensions were prepared from plate-grown bacteria resuspended in PBS to an optical density at 580 nm (OD_580_) of ~ 1.4. Twenty-five μL of this suspension (for *Bt* DW503; corresponding to a MOI of 1:40) or a ten-fold dilution of this suspension (for *Bm* ATCC23344; corresponding to a MOI of 1:4) were used to infect human monocytes. Bacteria-monocyte contact was promoted via centrifugation at 400 × g for 4 min. For assays with *Bt* DW503, MM6 monocytes were infected for 2 h, washed, and subsequently treated with 50 μg/mL of kanamycin (MP Biomedicals) for an additional 2 h to eliminate extracellular bacteria. Monocytes were again washed to remove residual antibiotic. Subsequently, a subset of monocytes was lysed with 0.5% saponin for 5 min at 37 °C. These monocyte lysates were then serially diluted, plated onto LB-agar plates, and incubated at 37 °C for 48 h. The remaining subset of monocytes was re-plated with complete medium for an additional 6 h of incubation at 37 °C in a 5% CO_2_ atmosphere. This subset of monocytes was then washed, lysed with saponin, and resulting lysates were serially diluted, plated, and incubated for 48 h at 37 °C. For assays with *Bm* ATCC23344, a similar protocol was followed with some modifications (1 h infection, 1.5 h kanamycin treatment, 7.5 h of additional incubation for a subset of monocytes, plating on Brucella-agar medium). For both assays, following incubation of plated lysates for 48 h at 37 °C, colony forming units (CFUs) representative of bacteria internalized by monocytes following an initial infection phase and kanamycin treatment (phagocytosis) or internalized by monocytes that underwent an additional 6 h (*Bt*) or 7.5 h (*Bm*) of incubation (replication) were enumerated. Intracellular growth indices were subsequently determined by dividing the CFUs representative of bacteria internalized at the replication time point by the CFUs representing internalization during phagocytosis.

### Gene expression

Transcriptome profiling of MM6 cells infected with *Bm* ATCC23344 or *Bt* DW503 was conducted in the context of the aforementioned intracellular survival assay. To capture differential expression of host monocyte genes during infection, the transcriptome of infected MM6 monocytes was assessed at 1 h, 3 h, or 6 h postinfection and compared to that of uninfected MM6 monocytes incubated for 1 h or 6 h. At the indicated time points postinfection (1, 3, or 6 h) or post-incubation (1 h or 6 h), independent quintuplicate sets of infected or uninfected MM6 monocytes were washed and centrifuged at 2400 × g for 3 min prior to RNA extraction. MM6 monocyte pellets were then resuspended in lysis buffer RLY (Isolate II RNA Mini Kit, Bioline, Inc.) supplemented with β-mercaptoethanol, vigorously vortexed, and lysates were stored at − 80 °C until further processing. Total RNA was extracted from monocyte lysates using Isolate II RNA Mini Kit (Bioline) according to manufacturer’s instructions. Quantification of RNA yields and 260/280_nm_ and 260/230_nm_ absorbance ratios were assessed using NANODROP 2000 Spectrophotometer (Thermo Scientific), and RNA integrity was evaluated via agarose gel electrophoresis. Out of the quintuplicate monocyte-derived total RNA samples representative of each condition, quadruplicate total RNA subsets with 260/280_nm_ and 260/230_nm_ absorbance ratios ≥2.0 were included in the microarray analysis. Subsequently, cDNA was synthesized from these total RNA subsets and labeled using the GeneChip Whole Transcriptome PLUS Reagent kit according to Affymetrix protocols. Ten μg of labeled and fragmented cDNA were then hybridized onto GeneChip Human Transcriptome 2.0 arrays for 16 h at 45 °C. Following washing and staining with Affymetrix Fluidics Station 450, arrays were scanned using the Affymetrix GeneChip Scanner 3000 7G Plus. The quality of hybridization was determined using internal controls present within the GeneChip Human Transcriptome Array 2.0.

### Microarray analysis

The data obtained across ~ 70,000 probe sets present within the GeneChip Human Transcriptome Array 2.0 were reduced to 26,831 probe sets with a corresponding gene entrez ID. Following normalization of the dataset using Robust Multichip Average, as implemented in Partek® software (Partek Inc., St. Louis, MO, USA), unsupervised and supervised analyses were performed. For the unsupervised analysis, the probe sets whose signals varied the most across the dataset were first determined by calculating the coefficient of variance (CoV). Probe sets that exceeded a CoV of 0.5 were then subjected to principal component analysis and hierarchical clustering to visualize the distribution of the dataset and determine the extent of a characteristic host cell response within each of the treatment groups. To avoid introducing uncontrolled variables into our experimental design, we used MM6 monocytes of the same passage subjected to identical experimental conditions, reagent preparations, and RNA isolation protocols. Following the initial assessment of the human MM6 monocyte response to each treatment condition, a supervised analysis, employing a univariate F tests with a random variance model, was conducted using BRB Array Tools (developed by Dr. Richard Simon and the BRB-ArrayTools Development Team) to investigate gene expression differences between *Bm-* or *Bt*-infected monocytes as compared to uninfected monocytes. Leave-one-out cross-validation (LOOCV) studies were then performed to assess the reproducibility of the datasets and test the abilities of the significant probe sets to truly distinguish between the classifiers (treatment groups). In these LOOCV studies, each array was left out in turn and a classifier was derived for the eight treatment groups by selecting probe sets significant at *p* < 0.001. The significant probe sets were then used with the 1st and 3rd nearest neighbor prediction model to predict the class identity of the array that was left out and not included when the classification model was constructed. Fold changes were calculated between *Bm-* or *Bt*-infected and uninfected monocyte groups only for those genes significant at *p* < 0.001 and exhibiting 100% correct classification by LOOCV.

### Gene ontology

To mine the array data for biologically relevant information regarding potential infection-driven immune response pathway regulation, probe sets differentially impacted at a significance level of *p* < 0.001 were used to populate experimentally observed human canonical pathways using Ingenuity Pathway Analysis software (IPA; Qiagen, http://www.ingenuity.com). Statistically significant canonical pathways were identified by Fisher’s exact test (*p <* 0.05) or z-score, with values of 2 < z < − 2 considered significant.

### Real-time quantitative PCR

Real time-quantitative PCR (qPCR) was performed on the StepOne (ThermoFisher Scientific) with the same total RNA samples from monocytes infected with *Bm* (1,3,6 h), *Bt* (1,3,6 h), or uninfected (1,6 h) analyzed by microarray, to validate the observed changes in expression of the human PTX3 gene. Total RNA was converted to cDNA using SensiFAST cDNA Synthesis Kit (BIO-65053, Bioline), according to manufacturer’s instructions, and qPCR was conducted using 2 μl of cDNA, Taqman Gene Expression Master Mix (4,369,016, Applied Biosystems), and IDT primer-probe sets in a final volume of 25 μl. The human GAPDH gene was used as the reference gene for normalization given its previously demonstrated stable expression in this cell line. This validation experiment was conducted in triplicates per each condition. Primer-probe sets are described in Table 1.

**Table 1:**
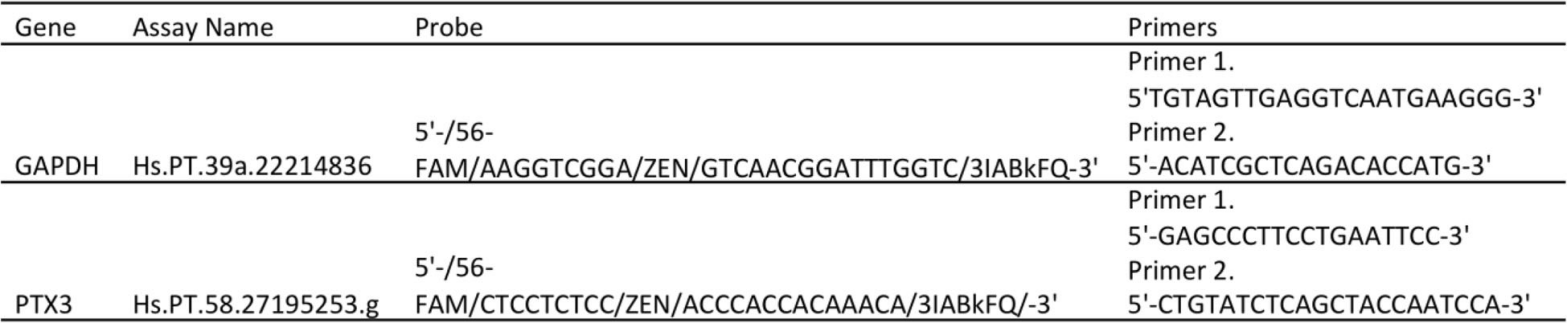
List of oligonucleotides used in real-time qPCR experiments

### Binding assays

These assays were conducted to characterize a potential binding interaction between PTX3 and *Bt* or *Bm.* The closely-related pathogen, *Bp*, was also included as a comparison. For assays with *Bt* DW503, plate-grown bacteria were resuspended in PBS to an OD_580_ of ~ 1.4. Ten microliters of this bacterial suspension were added to HBSS (10-508F, Lonza) containing 10 μg/mL of carrier-free recombinant human PTX3 (rhPTX3) (1826-TS/CF, R&D Systems) in a total volume of 100 μL. Following brief vortexing and centrifugation, tubes were incubated for 1 h in 5% CO_2_ at 37 °C to promote *Bt*-PTX3 binding. Thereafter, tubes were centrifuged at 1500 × g for 10 min to remove unbound rhPTX3. Bacterial pellets were then resuspended in HBSS containing 1% BSA Fraction V (Fisher Bioreagents) and 0.5 μg/mL of biotinylated, polyclonal, anti-human PTX3 antibody (BAF1826, R&D Systems). Tubes were briefly mixed, centrifuged, and placed at 4 °C for 30 min. Following washing with HBSS, bacterial pellets were resuspended in HBSS with 1% BSA and 10 μL of phycoerythrin (PE)-labeled streptavidin (F0040, R&D Systems). After brief mixing and centrifugation, tubes were subsequently placed at 4 °C for 30 min. Next, bacterial pellets were washed with HBSS, resuspended in PBS containing 2% paraformaldehyde, and incubated at room temperature for 20 min. Following a final wash, bacterial pellets were resuspended in 200 μL of PBS, and samples were assayed on the BD LSRII flow cytometer. To detect binding between PTX3 and *Bm* ATCC23344 or *Bp* K96243, modifications to the described experimental approach included incubation with biotinylated anti-hPTX3 antibody or phycoerythrin-labeled streptavidin for 1 h at room temperature, with all centrifugation steps conducted at ~ 14,000 × g for 5 min. Three negative controls were included for each assay and consisted of HBSS containing bacteria only, bacteria with anti-human PTX3 antibody and streptavidin-PE, and bacteria with rhPTX3 and streptavidin-PE. In parallel, *Pseudomonas aeruginosa* PA01 (a generous gift from Dr. Balazs Rada) was included as a positive control, as this strain has been previously reported to bind to rhPTX3 [23].

### Bactericidal assays with PTX3 and normal human serum

These assays were designed to identify whether a cooperative relationship may exist between PTX3 and the host complement system in potentially enhancing complement-directed lysis of *Burkholderia* sp., or, conversely, if bacteria may benefit from interactions with PTX3 via masking of critical surface components and evasion of complement binding and resulting lysis. To develop these assays, we utilized *Bt* DW503 wherein plate-grown bacteria were resuspended in PBS to an OD_580_ of ~ 1.4, and 10 μL of this suspension were added to tubes containing HBSS with 10 μg/mL of rhPTX3 (PTX3-bound bacteria) or HBSS alone (unbound control) in a total volume of 100 μL. Following brief vortexing and centrifugation, tubes were incubated for 1 h in 5% CO_2_ at 37 °C to allow binding between *Bt* and PTX3. Tubes were subsequently centrifuged at 1500 × g for 10 min to remove unbound rhPTX3 from PTX3-coated bacteria. Pooled normal human serum (NHS, IPLA-SER, Innovative Research) was then added as a source of active complement at different concentrations (5 to 50%) to HBSS containing rhPTX3-bound bacteria or unbound bacteria, in a total volume of 100 μL. A negative control consisting of HBSS and bacteria in the absence of NHS was also included. Following the addition of pooled NHS, all tubes (including no serum control) were subsequently incubated in 5% CO_2_ at 37 °C for 0 h, 2 h, or 6 h to assess for changes in bacterial CFUs reflective of differential complement-mediated lysis over time. At the indicated time points, PTX3-bound bacteria with serum, unbound bacteria with serum, and a *Bt*-no serum control were subjected to serial dilutions and plating for enumeration of bacterial CFUs after 48 h of incubation in 5% CO_2_ at 37 °C. Additional assays were performed with modification to the described experimental approach that included co-incubating *Bt* with 50% NHS and different concentrations of PTX3 (0.5 μg/mL, 1 μg/mL, or 5 μg/mL) prior to evaluating CFUs over time. An additional, in-parallel, comparative study was also undertaken with a distinct, unencapsulated, gram-negative bacterium, *Moraxella catarrhalis.* Serum-sensitive (*M. catarrhalis* O35E.2) and serum-resistant (wild-type (WT) *M. catarrhalis* O35E) strains [32] were included in these studies given a reported possible link between *M. catarrhalis* and PTX3 [33]. Following the above described method, mutant and WT *M. catarrhalis* strains were either pre-coated with 10 μg/mL PTX3 prior to the addition of 10% NHS or co-incubated with PTX3 and NHS before evaluating bacterial CFUs across time. For all assays, the bactericidal activity of complement in NHS was validated using the serum-sensitive *M. catarrhalis* O35E.2 strain.

### Bactericidal assays with PTX3, normal human serum, and opsonic antibodies

To investigate the interaction between bacteria and PTX3 in the context of an amplified classical complement pathway that may help drive the downstream lytic phase of complement, we aimed to enhance classical complement pathway activation by supplementing NHS with hyperimmune murine serum generated by vaccinating mice with *B. mallei* ATCC 23344 *batA* mutant strain [34]. The hyperimmune serum and purified IgG antibodies were shown to be opsonic to WT *Bm* as well as promote enhanced phagocytosis and intracellular killing of opsonized *Bm* by murine macrophages, when used at a concentration of 1:100 [34]. In separate assays, we demonstrated that antibodies in the hyperimmune serum are also able to recognize *Bt* at this concentration (data not shown). Given this, we hypothesized that enhanced antigen-antibody complex formation resulting from the addition of both NHS and hyperimmune murine serum may augment the activation of the classical complement pathway and, potentially, drive downstream lysis. For these assays, a similar methodology was followed as described in prior bactericidal assays, with differential survival kinetics of PTX3-bound or unbound *Bt* evaluated in 10% or 25% NHS supplemented with hyperimmune murine serum at a concentration of 1:100. As before, bacterial CFUs were assessed at 0, 2, and 6 h post-serum exposure.

### Intracellular survival assays in human monocytes with unbound or PTX3-bound Bt

Pentraxin-3 possesses opsonophagocytic properties towards several microbial agents and has additionally been reported to enhance the antimicrobial activity of host phagocytes towards opsonized agents [22, 23, 25–27, 31]. Intracellular survival assays were employed to determine if PTX3 pre-opsonization may enhance phagocytic uptake and killing of phagocytized bacteria, or, conversely, if binding of PTX3 to bacterial surfaces would promote cellular invasion and increased intracellular growth of opsonized bacteria. These assays evaluated the effect of PTX3 pre-opsonization on phagocytic uptake and replication of *Bt* (as a model for *Bm*) within human monocytes. Accordingly, 10 μL of a *Bt* DW503 suspension at an OD_580_ of ~ 1.4 were added to HBSS containing 10 μg/mL of rhPTX3 (PTX3-bound bacteria) or HBSS alone (unbound *Bt* control) in a total volume of 100 μL. Binding of *Bt* to rhPTX3 was promoted via incubation in 5% CO_2_ at 37 °C for 1 h. Following incubation, *Bt*-hPTX3 complexes or unbound *Bt* in HBSS were then subjected to centrifugation at 1500 × g for 10 min. PTX3 pre-bound or unbound *Bt* were then resuspended in 25 μL of complete medium and used to infect two, quadruplicate sets of 1 × 10^6^ human MM6 monocytes/mL, respectively. Intracellular survival assays were performed as previously described, with bacteria enumerated at 4 h (phagocytosis) or 10 h (replication) postinfection. Bacterial CFUs representative of phagocytosis and overall intracellular bacterial growth indices were compared between PTX3 pre-bound and unbound conditions. Additional assays were conducted in the presence of NHS to characterize the interaction between PTX3 pre-coating of bacterial surfaces and active complement on the outcome of *Bt* infection of human monocytes. Two, quadruplicate sets of 1 × 10^6^ human monocytes/mL were infected with PTX3 pre-bound or unbound *Bt*, respectively, in the presence of 10% NHS. At 4 h (phagocytosis) or 10 h (replication) postinfection, bacteria were enumerated and CFUs representative of phagocytosis and overall intracellular bacterial growth indices were compared between PTX3 pre-bound and unbound conditions. As in prior assays, the bactericidal activity of complement in NHS was validated using the serum-sensitive *M. catarrhalis* O35E.2 strain for all assays.

### Opsonophagocytic assays

These assays were designed to assess the impact of PTX3 pre-opsonization of bacteria on phagocytic uptake by human monocytes at an earlier time point more representative of the phagocytic process (ie., 30 min postinfection). For these assays, both the previously used PTX3 concentration (10 μg/mL) and a higher concentration of PTX3 (30 μg/mL) were included to evaluate possible concentration-dependent effects on opsonophagocytosis. *Bt* was utilized for these assays wherein 10 μL of a suspension at an OD_580_ of ~ 1.4 were added to HBSS containing 10 μg/mL or 30 μg/mL of rhPTX3 (pre-coated *Bt*) or HBSS alone (unbound *Bt* control) in a total volume of 100 μL. Binding of *Bt* to rhPTX3 was promoted via incubation in 5% CO_2_ at 37 °C for 1 h. Following incubation, *Bt*-hPTX3 complexes or unbound *Bt* in HBSS were then subjected to centrifugation at 1500 × g for 10 min. PTX3 pre-coated or unbound *Bt* were then resuspended in 25 μL of complete medium and used to respectively infect two, duplicate or triplicate sets of 1 × 10^6^ human MM6 monocytes/mL seeded in the presence of 10% NHS. Following 30 min of infection (phagocytosis), monocytes were subjected to centrifugation at 400 × g for 4 min, washed with complete medium, and resuspended in 0.5% saponin for 5 min in 5% CO_2_ at 37 °C. Resulting monocyte lysates were serially diluted and plated onto LB-agar plates to assess the phagocytic uptake of PTX3-*Bt* or *Bt* by human monocytes within 30 min of infection.

## Results

### Intracellular survival assays

**Fig. 1:**
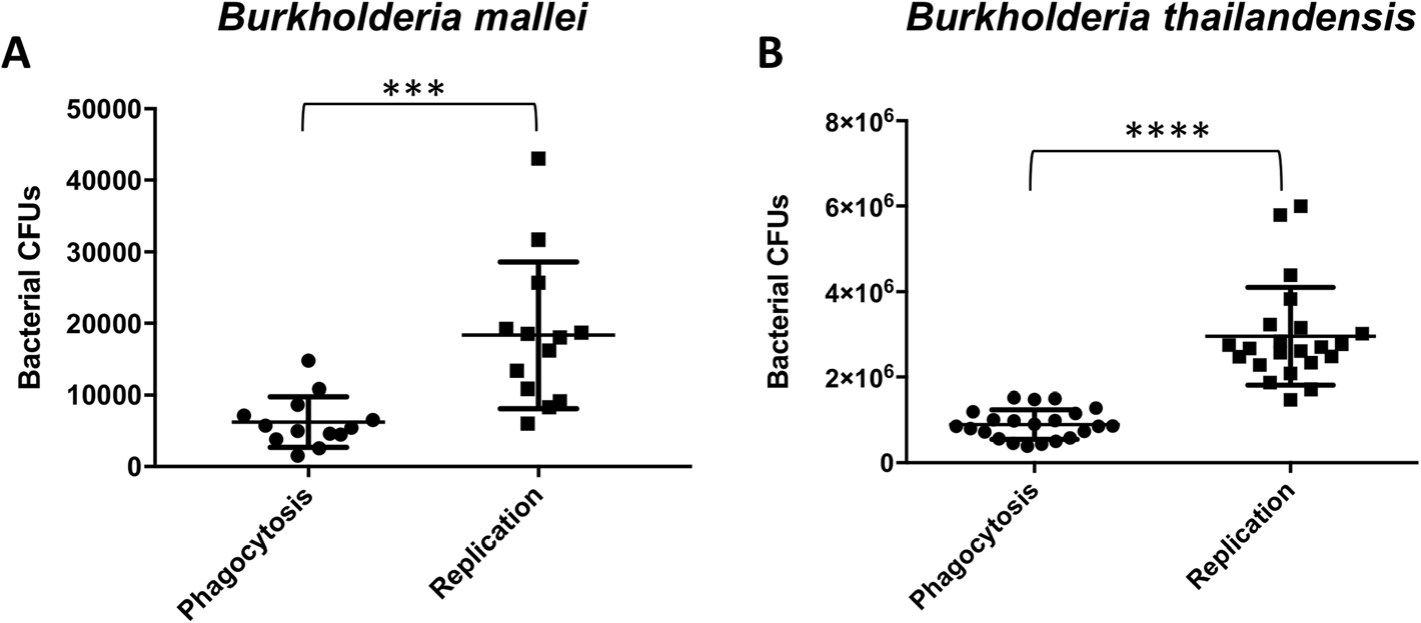
*Bm* and *Bt* replication in human MM6 monocytes. **a** *Bm* exhibits significant intracellular growth within human monocytes between phagocytosis (2.5 h postinfection) and replication (10 h postinfection) time points (unpaired t-test***p = 0.0005). **b** *Bt* exhibits significant intracellular growth within human monocytes between phagocytosis (4 h postinfection) and replication (10 h postinfection) time points (unpaired t-test ****p < 0.0001)

The results from intracellular survival assays performed with *Bm* ATCC23344 or *Bt* DW503 and human MM6 monocytes demonstrate that *Bm* or *Bt* replicate significantly within human monocytes (***p = 0.0005 or ****p < 0.0001, respectively). Intracellular growth indices of *Bm* or *Bt* in these human monocytes are comparable and average ~ 3.2 and ~ 3.5, respectively (Fig. 1). Thus, a ten-fold dilution of *Bm* suspension at an OD_580_ of ~ 1.4 replicated within human monocytes to the same rate as an undiluted suspension of *Bt*. This finding suggests differential modulation of monocyte gene expression and/or may be related to pathogenic mechanisms specific to *Bm*.

### Microarray analysis

A hierarchical clustering [35] of genes with a coefficient of variation greater than 0.5 showed that quadruplicate monocyte subsets representative of each treatment group (Table 2) clustered together, indicating that each treatment elicited a specific and distinct transcriptome profile in human MM6 monocytes (data not shown). Then, a supervised analysis used to identify genes differentially expressed between the treatment groups identified 9491 probe sets (corresponding to 9013 genes) as significant (*p* < 0.001) out of 26,831 probe sets tested for significance. Leave-one-out cross-validation studies were then performed to test the abilities of these significant probe sets to truly distinguish between the treatment groups. Overall, with eight treatment groups, by chance alone one would expect an error rate of 87.5%. In our study, the classifiers performed flawlessly and correctly predicted the treatment groups with 100% accuracy. This finding indicates that gene expression differences can be used to distinguish between the treatment groups with a high level of confidence.

**Table 2:**
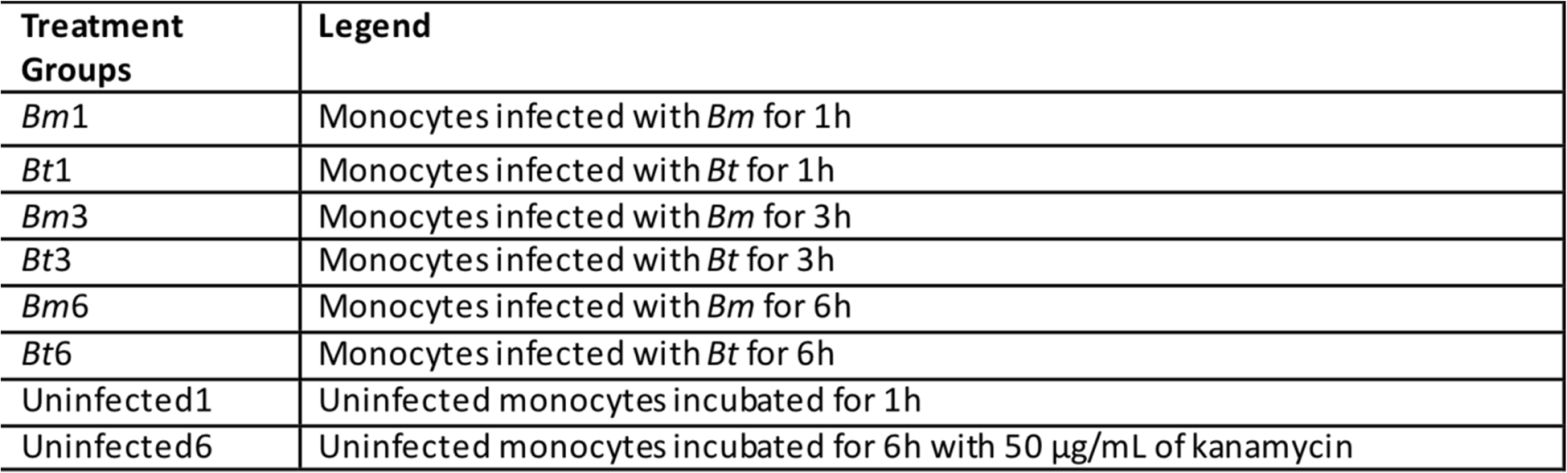
Gene expression profile conditions for infected or uninfected human MM6 monocytes

### Canonical pathway and immune response-related gene analyses

**Fig. 2:**
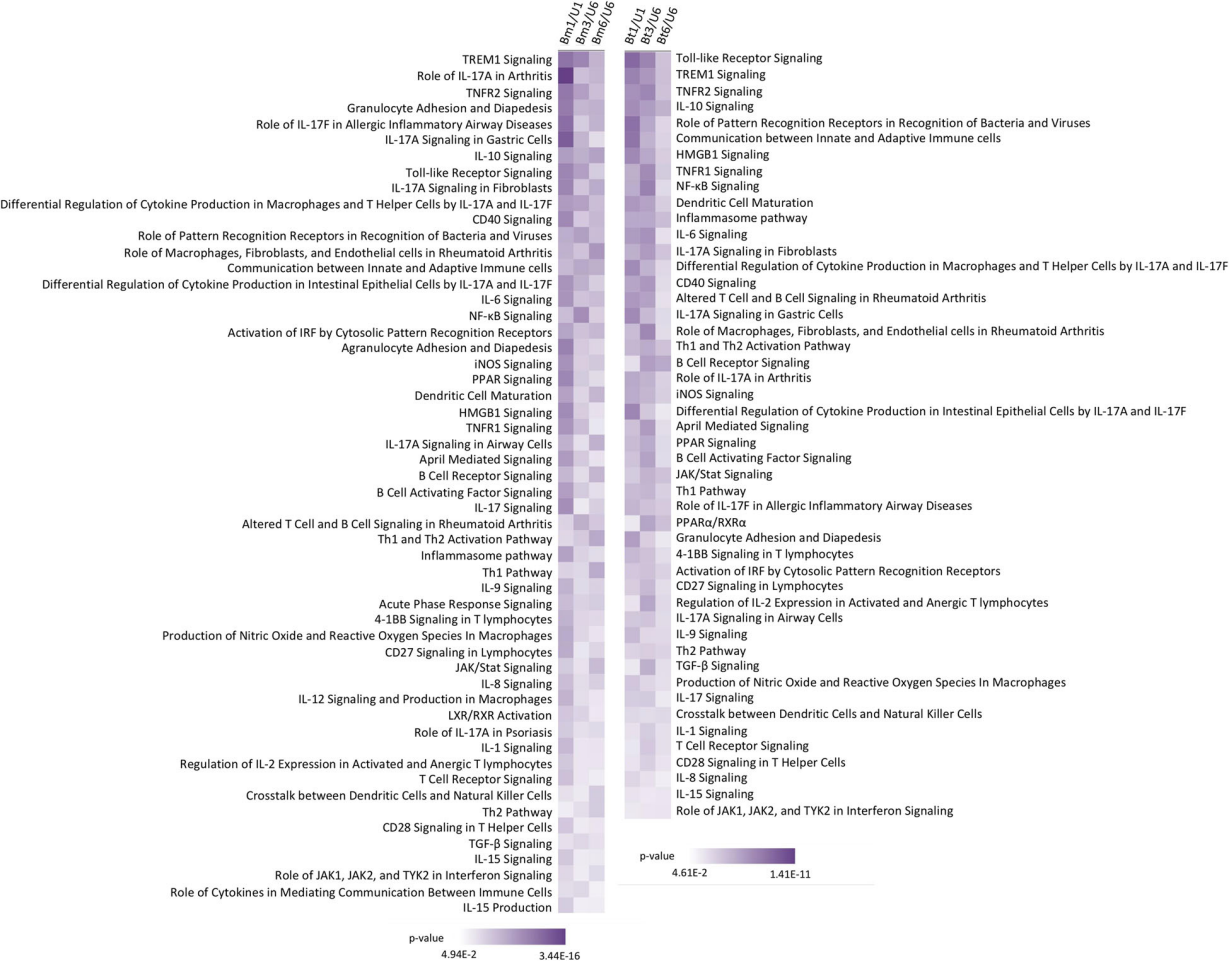
Canonical pathways significantly enriched in human monocytes infected with *Bm* or *Bt*. Numerous immune response-related pathways are significantly regulated during *Bm* (3.44 × 10^− 16^ > *p ≤* 4.94 × 10^− 2^) or *Bt* (1.41 × 10^− 11^ > *p ≤* 4.61 × 10^− 2^) infection of human monocytes. *Bm*1/U1, *Bm*-infected human monocytes relative to uninfected monocytes incubated for 1 h; *Bm*3/U6, *Bm*-infected human monocytes for 3 h relative to uninfected monocytes incubated for 6 h; *Bm*6/U6, *Bm-*infected human monocytes relative to uninfected monocytes incubated for 6 h; *Bt*1/U1, *Bt*-infected human monocytes relative to uninfected monocytes incubated for 1 h; *Bt*3/U6, *Bt*-infected human monocytes for 3 h relative to uninfected monocytes incubated for 6 h; *Bt*6/U6, *Bt*-infected human monocytes relative to uninfected monocytes incubated for 6 h

**Fig. 3:**
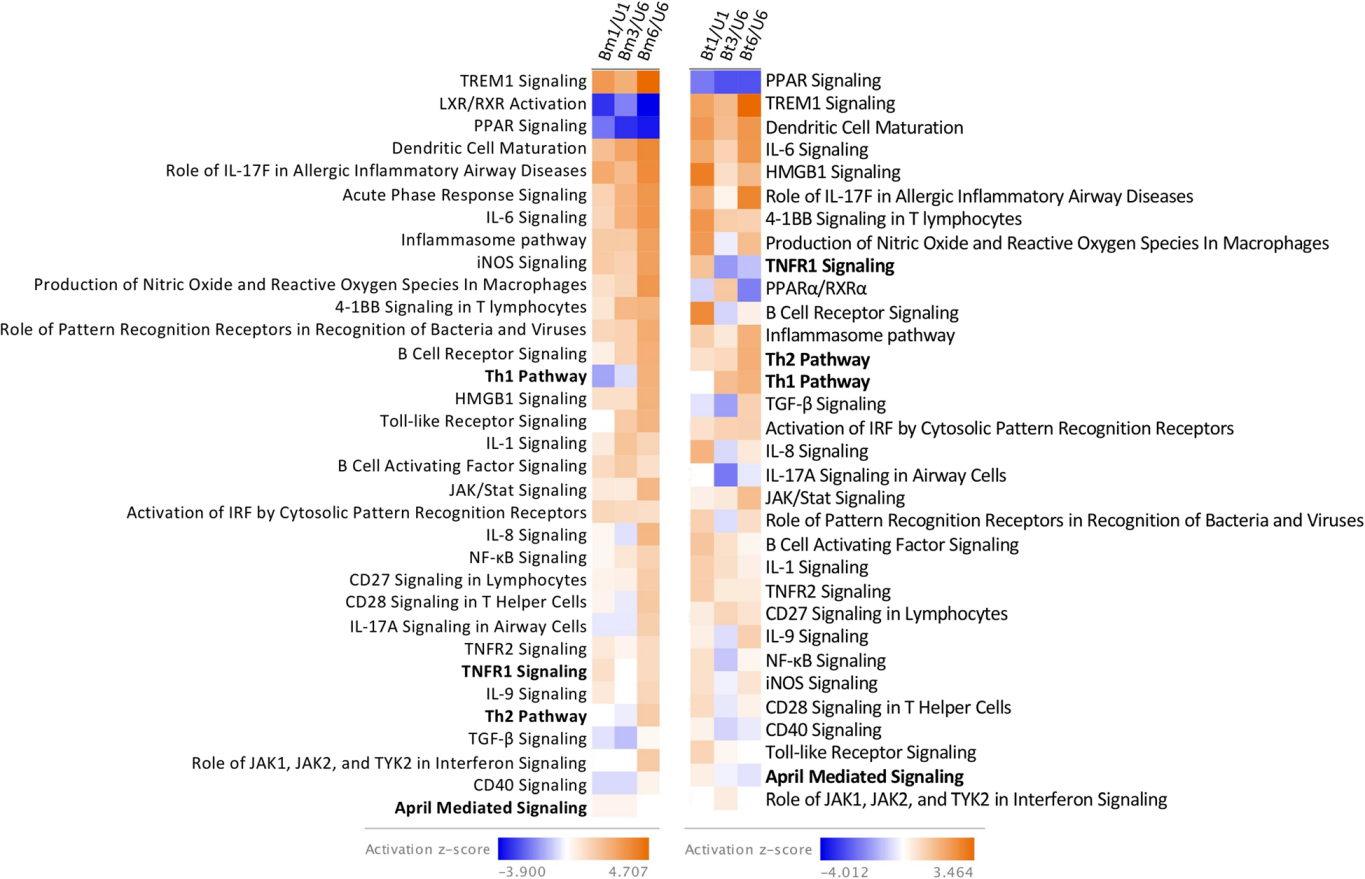
Differential expression of immune response-related canonical pathways during infection of human monocytes with *Bm* or *Bt*. Among significantly enriched immune response-related pathways (*p <* 0.05), several pathways are significantly upregulated or downregulated during *Bm* or *Bt* infection of human monocytes at individual or all time points (1, 3, 6 h). Highlighted in bold are pathways that differ between *Bm* or *Bt* infections across two time points. *Bm*1/U1, *Bm*-infected human monocytes relative to uninfected monocytes incubated for 1 h; *Bm*3/U6, *Bm*-infected human monocytes for 3 h relative to uninfected monocytes incubated for 6 h; *Bm*6/U6, *Bm-*infected human monocytes relative to uninfected monocytes incubated for 6 h; *Bt*1/U1, *Bt*-infected human monocytes relative to uninfected monocytes incubated for 1 h; *Bt*3/U6, *Bt*-infected human monocytes for 3 h relative to uninfected monocytes incubated for 6 h; *Bt*6/U6, *Bt*-infected human monocytes relative to uninfected monocytes incubated for 6 h

**Fig. 4:**
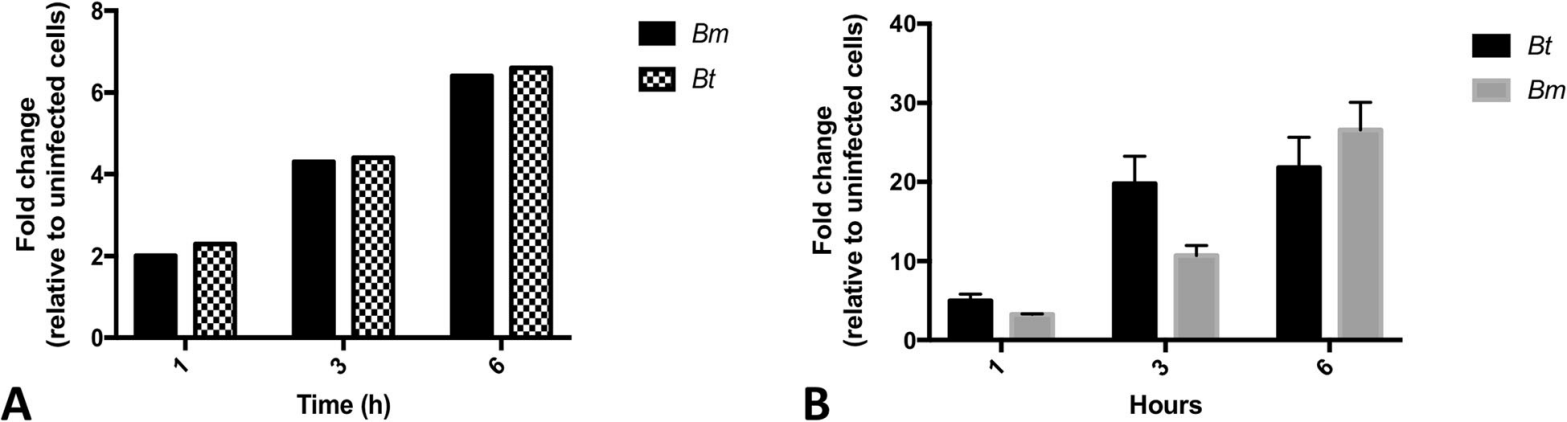
Human monocytes upregulate expression of the soluble pattern recognition receptor, PTX3, during infection with *Bm* or *Bt*. **a**. Pentraxin-3 gene expression significantly increases in a time-dependent manner, with highest expression at 6 h postinfection (*p* < 0.001). Each condition was assessed in quadruplicates. **b**. Real-time quantitative PCR analysis of the PTX3 gene found to be differentially expressed by microarray analysis. Each condition was assessed in triplicates

Ingenuity pathway analysis demonstrated significant enrichment and modulation of several immune response-related pathways (*p <* 0.05). Among these, the Pattern Recognition Receptor (PRR) pathway was significantly expressed in human monocytes during infection with *Bm* (*p <* 3.96 × 10^− 6^) or *Bt* (*p <* 8.32 × 10^− 4^) (Fig. 2). Analysis of this pathway by z-score showed upregulation during infection of human monocytes with *Bm* or *Bt*, although statistical significance was only reached for *Bm*-infected monocytes (z = 2.646) (Fig. 3). When further mining the array data, at the gene level, for evidence of modulation of monocyte expressed members of the PRR pathway, the soluble PRR, pentraxin-3 (PTX3) was found to exhibit the highest gene expression fold change in infected human monocytes, with significantly induced expression of this gene across time (*p <* 0.001) (Fig. 4a). This notable, time-dependent, induction in monocyte PTX3 expression during *Bm* or *Bt* infection was validated by qPCR (Fig. 4b) as up- or down-regulated, albeit with magnitudes different from those recorded by microarray analysis.

### Interaction between human PTX3 and *Bt* or *Bm* during innate immune responses

**Fig. 5:**
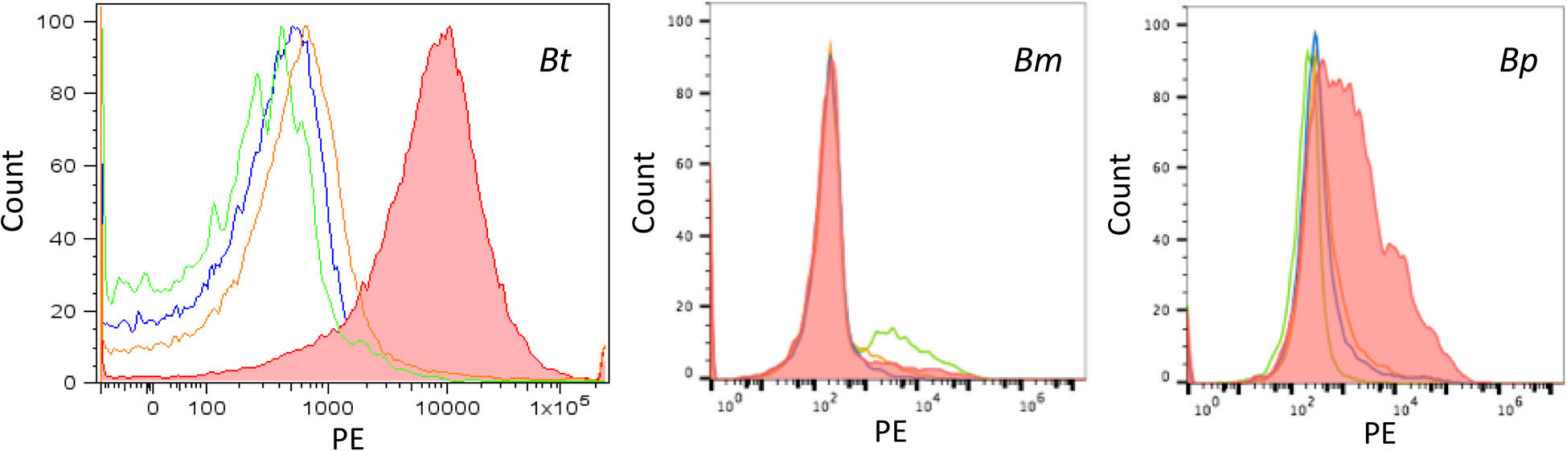
Human PTX3 recognition of select *Burkholderia* sp. Binding between rhPTX3 and 10^6^–10^7^ bacteria was demonstrated via biotinylated anti-hPTX3 antibody and PE-labeled streptavidin. Control fluorescence is represented by the green (bacteria only), blue (bacteria, rhPTX3, streptavidin-PE), orange (bacteria, anti-hPTX3 antibody, streptavidin-PE) curves. The data are representative of at least *n* = 2 independent assays. The data across all treatment groups are normalized to the number of cells

Given that infected monocytes were found to exhibit progressive, time-dependent, enhanced expression of the soluble PRR, PTX3, by microarray and qPCR analysis, flow cytometry was then used to determine whether following its expression and production, PTX3 may target and bind *Bm* or *Bt* as part of the innate immune response. The closely-related pathogen, *Bp*, was also included in these assays. Flow cytometry data demonstrated recognition and binding of *Bt* and *Bp* by PTX3, with lack of binding of this PRR to *Bm* (Fig. 5). *Pseudomonas aeruginosa* (PA01) was used as a positive control for these assays and binding to PTX3 was demonstrated as previously described (data not shown).

### Characterizing the role of PTX3 binding in the innate immune response against *Bt*

#### The role of PTX3 and host complement system during infection with Bt

**Fig. 6:**
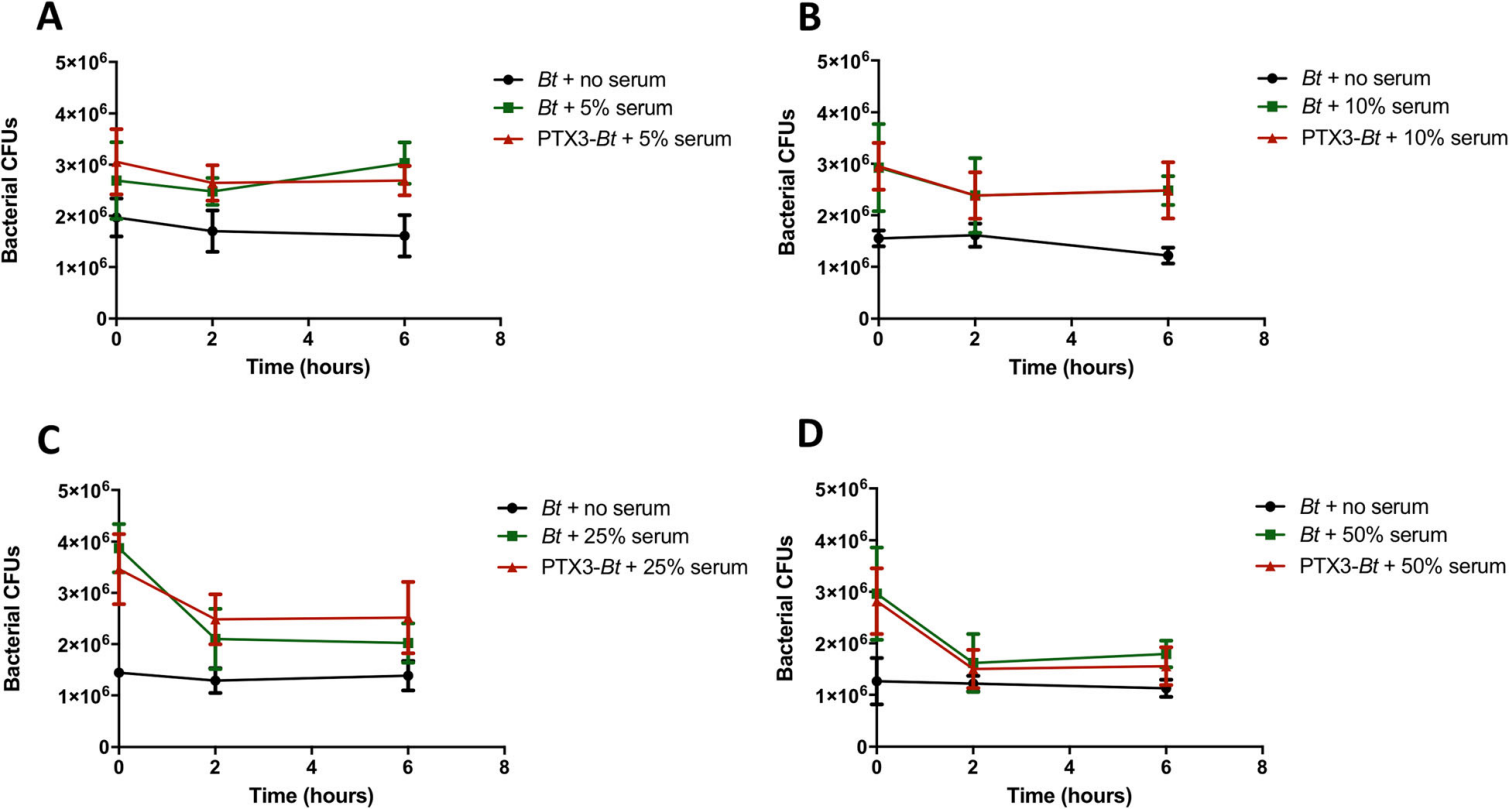
Human PTX3 does not potentiate the lytic activity of complement towards *Bt*. Complexes of PTX3 and 10^7^ *Bt* were subjected to (**a**) 5%, (**b**) 10%, (**c**) 25%, or (**d**) 50% NHS, and bacterial CFUs were enumerated at 0, 2, or 6 h post-normal human serum exposure. Two-way ANOVA and Bonferroni multiple comparisons revealed no significant differences in CFUs between PTX3-bound or free bacteria. Each dataset is representative of *n* = 4 independent experiments

The novel binding interaction between PTX3 and *Bt* or *Bp* prompted an investigation of the potential biological functions of PTX3 as part of the innate immune response. Through serum bactericidal assays with *Bt*, we sought to define whether binding to PTX3 may represent a host-related defense mechanism or may be promoted by bacteria as an immunoevasive strategy. Specifically, the aim of these assays was to determine if PTX3 recognition of bacteria may represent part of the serum-based innate immune defenses towards achieving complement-mediated bacterial lysis, or, conversely, if bacteria may benefit from coating with PTX3 via masking of critical surface components to evade complement deposition and lysis. Four, independent serum bactericidal assays performed for each tested serum concentration (5, 10, 25, 50%) demonstrate that PTX3-bound *Bt* or free *Bt* and are similarly resistant to complement-mediated lysis in a 6 h period (even at 50% NHS) (Fig. 6), which is consistent with prior studies with *Bt* [36, 37]. Thus, pre-coating with PTX3 does not alter the complement resistance of *Bt*, as there are no further observed reductions in CFUs for PTX3-bound bacteria across the tested NHS concentrations. In additional assays wherein *Bt* was co-incubated (rather than pre-incubated) with 0.5–5 μg/mL of PTX3 and 50% NHS for 6 h, *Bt* likewise exhibited similar resistance to NHS, and this serum resistance was not attenuated by PTX3 co-incubation (data not shown). As a comparison, in-parallel assays conducted with serum-sensitive *M. catarrhalis* O35E.2 and serum-resistant *M. catarrhalis* O35E strains similarly demonstrated that PTX3 pre-coating neither potentiated the bactericidal activity of 10% NHS against the serum-sensitive strain, nor did it act synergistically with complement to induce bactericidal activity against the serum-resistant WT strain, in a 2 h period (data not shown). Altogether, PTX3 was not observed to potentiate the terminal lytic phase of complement across two different bacterial species that included both serum-resistant (*Bt* DW503, *M. catarrhalis* O35E) and serum-sensitive (*M. catarrhalis* O35E.2) strains. For all assays, the bactericidal activity of complement in NHS was validated by demonstrating consistent killing of *M. catarrhalis* O35E.2 following incubation with 10% NHS for 2 h (data not included).

**Fig. 7:**
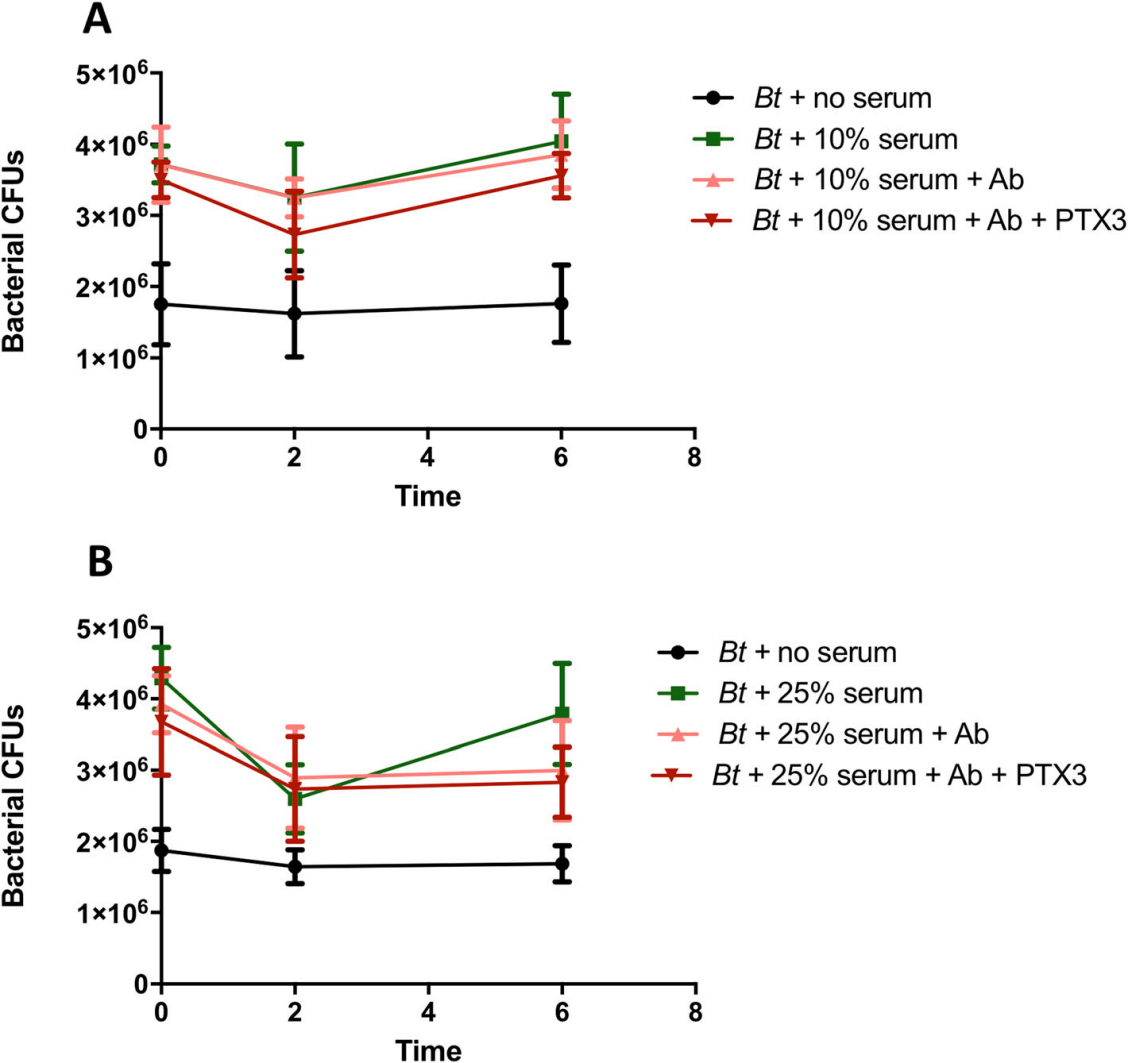
Opsonic antibodies fail to synergize with PTX3 in bolstering the lytic activity of complement towards *Bt*. Complexes of PTX3 and 10^7^ *Bt* were subjected to (**a**) 10% or (**b**) 25% NHS +/− hyperimmune serum containing anti-*Bm* antibodies (Ab) also opsonic to *Bt*. Bacterial CFUs were enumerated at 0, 2, or 6 h post-serum exposure. Two-way ANOVA and Bonferroni multiple comparisons revealed no significant differences in CFUs between PTX3-bound or free bacteria. Data are representative of four to five assays per serum concentration

To investigate the possibility that serum resistance displayed by *Bt* (Fig. 6) may be masking a synergistic relationship between complement and PTX3 towards bacterial lysis, further bactericidal assays were conducted in the context of an amplified classical complement pathway. Accordingly, NHS was supplemented with murine hyperimmune serum containing antibodies known for their opsonic activity for *Bm* and *Bt*. While anti-*Bm* antibodies within the hyperimmune serum are also opsonic to *Bt* and may amplify the initiation of the classical complement pathway, the presence of these opsonic antibodies at a concentration of 1:100 did not significantly impact the lytic phase of the complement cascade, with no significant decrease in the serum resistance of PTX3-bound or free *Bt* in our assays (Fig. 7).

#### The role of PTX3 and host complement system during monocytic infection with Bt

**Fig. 8:**
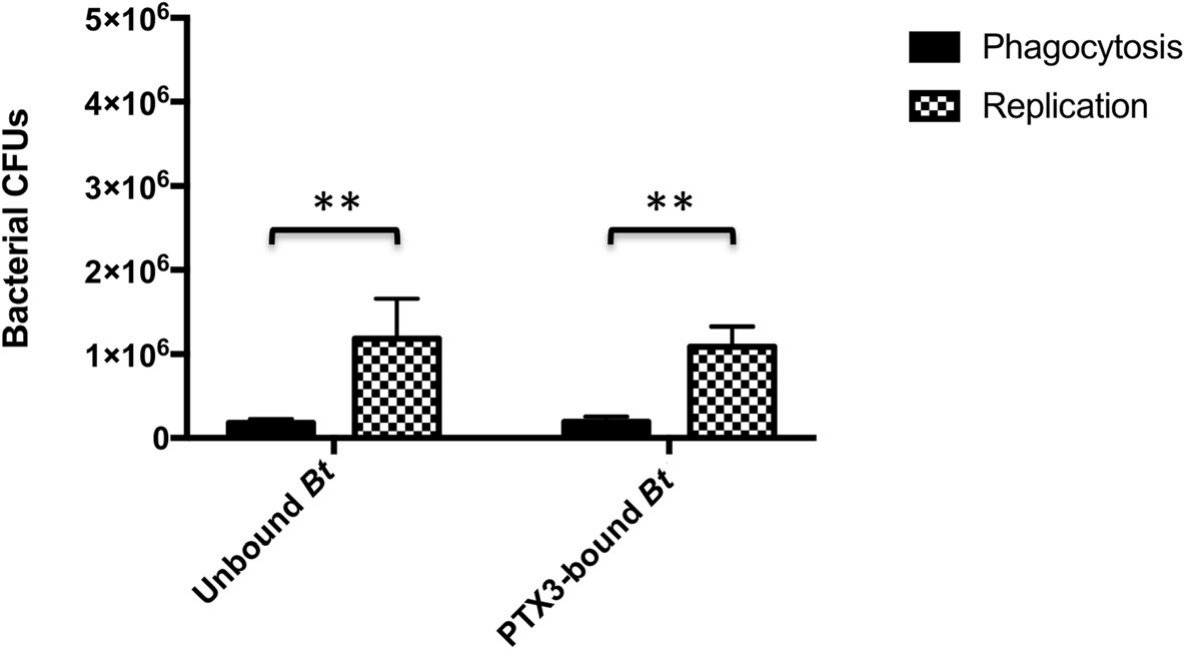
The impact of PTX3 on bacterial phagocytosis and replication within human monocytes. Pre-infection heterocomplexes of 10 μg/mL of PTX3 and 10^7^ *Bt* were used to infect 1 × 10^6^ human monocytes in the absence of complement. Intracellular bacterial CFUs were enumerated 4 h postinfection (phagocytosis) and 10 h postinfection (replication). PTX3-bound or unbound *Bt* did not exhibit significant differences in phagocytic uptake or replication within human MM6 monocytes (unpaired t-test). Both PTX3 pre-bound and unbound *Bt* displayed significant intracellular growth within human MM6 monocytes (**p = 0.0026, unpaired t-test)

Given the reported capacity for PTX3 opsonization to enhance both the phagocytosis and intracellular clearance of targeted agents [22, 23, 25–27, 31], we sought to determine if the capacity for PTX3 to recognize *Bt* may, instead, be involved in facilitating uptake by host monocytes or may influence the intracellular survival of *Bt* within these phagocytic cells. Specifically, we employed intracellular survival and opsonophagocytic assays to determine if PTX3 pre-opsonization may enhance phagocytosis and/or intracellular killing of opsonized *Bt*, or, conversely, if binding of PTX3 to bacterial surfaces promotes cellular invasion and augments intracellular growth of opsonized bacteria. Results from *n* = 3, independent, intracellular survival assays with *Bt* did not reveal a significant difference in phagocytosis or replication between PTX3 pre-bound or unbound bacteria, in human monocytes. In fact, similar intracellular growth indices were noted across both conditions (Fig. 8).

**Fig. 9:**
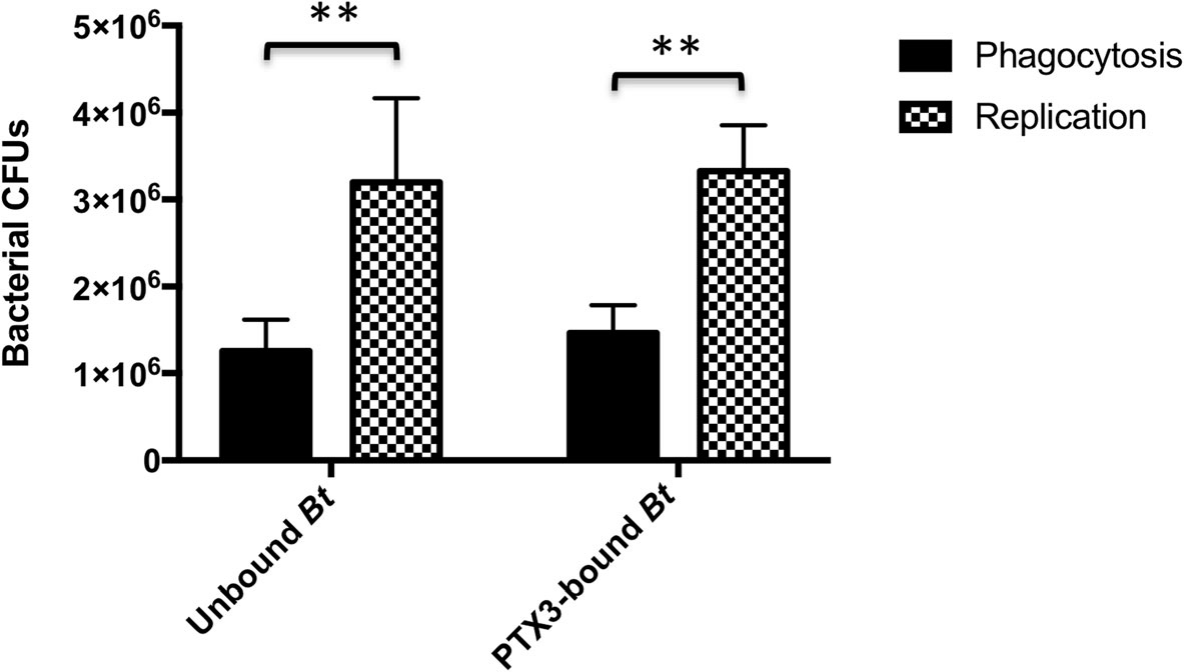
The role of PTX3-complement complexes in *Bt* intracellular growth within monocytes. Pre-infection complexes between 10 μg/mL of PTX3 and 10^7^ *Bt* were used to infect 1 × 10^6^ human monocytes in the presence of active complement (pooled NHS). Intracellular bacterial CFUs were enumerated 4 h postinfection (phagocytosis) and 10 h postinfection (replication). PTX3-bound or unbound *Bt* did not exhibit significant differences in phagocytic uptake or replication in human MM6 monocytes (unpaired t-test). Overall, both PTX3 pre-bound and unbound *Bt* displayed significant intracellular growth within human MM6 monocytes (**p = 0.0042, unpaired t-test)

The reported complement dependence of PTX3-mediated opsonophagocytosis of select organisms [23, 25, 26] suggested that the lack of an observable effect of PTX3 on phagocytosis or replication of *Bt* within human monocytes in our assays, may be directly related to the absence of an active source of complement in this in vitro infection system. However, additional intracellular survival assays (*n* = 4) conducted in the presence of pooled NHS (as a source of active compliment) likewise did not reveal a complement-dependent effect of PTX3 on phagocytosis, replication, or overall growth of *Bt* within MM6 monocytes (Fig. 9). These data then prompted us to evaluate the possibility that the lack of a discernible opsonophagocytic effect may be related to the time points at which phagocytosis was assessed in our assays (4 h postinfection). Given that phagocytosis of *Burkholderia* sp. can occur as early as 10–15 min postinfection [37, 38] and that PTX3-mediated effects on phagocytosis are predominantly observed within 15–30 min or up to 2 h postinfection [22, 23, 25–27], we surmised that PTX3-driven effects may, in fact, not be observable at time points that are far beyond the phagocytic process.

**Fig. 10:**
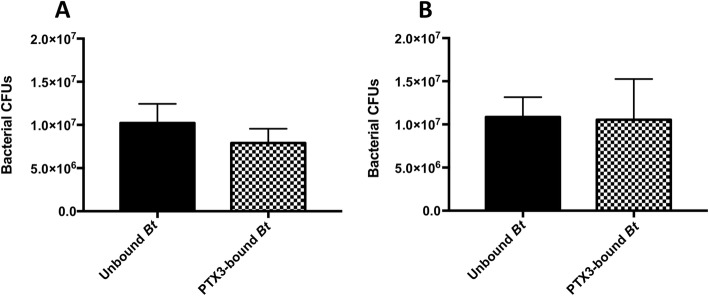
The role of PTX3-complement complexes in *Bt* phagocytosis by human monocytes. Pre-infection complexes between 10 μg/mL (**a**) or 30 μg/mL (**b**) of PTX3 and 10^7^ *Bt* were used to infect 1 × 10^6^ human monocytes in the presence of active complement (pooled NHS). Intracellular bacterial CFUs representative of phagocytosis were enumerated following 30 min of infection. An unpaired t-test did not reveal significant differences between phagocytosis for PTX3-pre-bound or unbound *Bt*. Data in (**a**) and (**b**) are each representative of *n* = 3 independent assays

Thus, we next aimed to evaluate a possible PTX3-mediated opsonophagocytic effect on *Bt* at an earlier time point more representative of phagocytosis (30 min postinfection). However, data from these additional assays likewise did not demonstrate a significant difference in the phagocytic uptake of PTX3-bound or non-PTX3 bound *Bt*, even at this earlier time point (Fig. 10a, b) or at a 3-fold higher PTX3 concentration (30 μg/mL) (Fig. 10b), which is in agreement with our 4 h phagocytosis data in prior assays (Figs. 8, 9).

## Discussion

Our studies are the first to develop an intracellular survival assay system to study the pathogenesis of *Burkholderia mallei* in the context of a human monocytic cell line. Following the establishment of this system, we report on human monocyte gene regulation by *Bm* (and the closely related *Burkholderia* sp., *Bt*) on a global, genome-wide scale, with evidence of modulation of several immune response-related genes and pathways. Of these, the Pattern Recognition Receptor (PRR) pathway was represented among significantly enriched canonical pathways in the transcriptome of *Bm*- or *Bt*-infected human monocytes, with evidence of pathway activation during both infections.

Currently, our knowledge of the relationship between members of the PRR pathway and *Bm* is limited to few interactions with receptor-based and soluble PRRs. Specifically, it is known that the *Bm* LPS can stimulate TLR4, resulting in the activation of NF-κB [6] and the release of IL-8 [39]. It has also been suggested that members of the complement system within host naïve serum may actively recognize and interact with *Bm*, resulting in enhanced phagocytosis by macrophages, as compared to the phagocytosis of *Bm* alone [9, 34, 40]. Given that the relationship between PRRs and *Bm* still remains poorly defined, we pursued this pathway for further study to explore innate immune system mediators involved in the recognition and coordination of pathogen clearance that may ultimately be harnessed as innovative *Burkholderia*-targeted immunotherapies.

Among the members of the PRR pathway, the soluble PRR, PTX3, was found to be highly upregulated by *Bm*- or *Bt*-infected human monocytes in a time-dependent manner, as evidenced by both microarray and qPCR analyses. Given that PTX3 has been shown to be involved in microbial recognition, complement activation and modulation, opsonophagocytosis, and host resistance against several agents [20–27, 29–31], we sought to elucidate the previously unexplored role of PTX3 in the context of *Burkholderia* sp., with a particular interest in its implications for *Bm* pathogenesis. Following upregulation of the soluble PRR, PTX3, by *Bm-* or *Bt*-infected monocytes, we report on the novel binding of PTX3 to environmental *Burkholderia* sp., *Bt* and *Bp*, together with the lack of binding to the host-adapted pathogen, *Bm*. It is possible that this binding interaction between PTX3 and *Bt* or *Bp* reflects part of the innate immune response against intravascular bacteria, while lack of recognition of *Bm* may suggest a possible evasive strategy by this bacterium. The binding (and thus recognition) of PTX3 by *Bt* and *Bp* (but not *Bm*) could be related to the fact that these two species are more closely genetically related [3, 41]. However, this explanation is not likely given that there is still 99% DNA sequence homology among conserved regions of *Bm* and *Bp* or *Bt* (as reviewed by [42]). Thus, the inability of PTX3 to target *Bm* may rather be related to the fact that *Bt* and *Bp* are environmental organisms, while *Bm* is a host-adapted bacterium [2, 4, 41]. Specifically, since *Bm* has adapted to evade host immune responses to allow for intracellular replication and long-term persistence within host tissues, it conceivable that this bacterium would have the inherent capacity to evade recognition and binding by a soluble PRR, such as PTX3, as a survival advantage. The possibility of PTX3 evasion by *Bm* contributing to enhanced survival of this bacterium within the host is worthy of exploration.

To address potential downstream effector mechanisms associated with PTX3 binding to *Bt* or *Bp*, follow-up studies explored the interplay between PTX3, *Bt,* and innate immune response components, such as complement and monocytes. Serum bactericidal assays conducted to investigate the relationship between complement, PTX3, and *Bt* did not identify a significant role for this PRR in amplifying the terminal lytic phase of complement towards this agent. Specifically, similar growth kinetics were observed for unbound *Bt*, PTX3 pre-bound *Bt,* or *Bt* co-incubated with PTX3 in NHS, with no apparent attenuation of *Bt* complement resistance imparted by PTX3 binding or co-incubation. These results suggested that PTX3-mediated potentiation of complement-directed lysis may be masked by the high level of serum resistance inherent to this bacterium and prompted additional bactericidal assays performed in the context of an amplified classical complement pathway using murine hyperimmune serum. However, these assays likewise showed a lack of discernible potentiation of bacterial lysis by PTX3. Possible reasons for apparent lack of potentiation may be due to the fact that hyperimmune serum at the concentration of 1:100 used in our assays may still not be sufficient to overcome the inherently high level of serum resistance exhibited by *Bt* at 10% or 25% NHS. Additionally, it is possible that PTX3 bound to bacterial surfaces does not cooperate with opsonic antibodies to promote detectable potentiation of the terminal lytic pathway of complement. Collectively, these global data obtained with NHS +/− hyperimmune serum suggest that while binding of PTX3 to *Bt* may reflect innate immune system recognition as part of the host effort to recruit complement proteins to the bacterial surface and target bacteria for lysis, that this bacterium may be resistant to PTX3-mediated enhancement of the lytic phase of complement. Alternatively, these data may instead reflect novel insights on PTX3 mechanisms of action in suggesting that the properties of this PRR are confined to the initiation of the complement pathway and recruitment of complement proteins to microbial surfaces [30, 31], rather than also regulating the activation of the terminal lytic phase. An apparent lack of involvement of PTX3 in complement-mediated lysis was also noted for two *Moraxella catarrhalis* strains in our study, as well as in published work by *Moalli* et al. 2011 with *Pseudomonas aeruginosa* [23]. Altogether these findings suggest that, perhaps, the role of PTX3 may truly be confined to the recruitment of complement to microbial surfaces, rather than also activating the lytic pathway. That being said, we cannot exclude the possibility that all three bacterial species may be capable of evading PTX3-mediated amplification of innate immune responses, with PTX3 production representing a futile host response to amplify complement-directed lysis and limit infection.

Beyond investigating the cooperative role of PTX3 with complement in the context of *Bt*, we further examined the impact of this PRR on the host monocyte-bacteria interface both in the absence and presence of active complement. In these assays, binding of *Bt* by PTX3 was not demonstrated to enhance the uptake (at early or later time points postinfection, or multiple PTX3 concentrations) or influence the replication of this bacterium in human monocytes. Although PTX3 recognition of bacteria may truly not play a significant role in the phagocytic uptake or replication of *Bt* within monocytes or in the context of the human MM6 monocytic cell line, we sought to clarify these results by conducting additional assays to determine the capacity for human PTX3 to bind to the surface of these monocytes. In *n* = 5, independent, flow cytometry assays, human PTX3 was not demonstrated to bind to the surface of human MM6 monocytes. For these assays, a polyclonal, biotinylated, human anti-CD14 antibody (BAF383, R&D Systems) was used as a positive control and consistently highlighted the surface expression of this marker in our cell line (data not shown). We also further validated the conditions of these assays by conducting, in-parallel, binding assays with *Bt* and PTX3 and demonstrating consistent binding as previously described. Altogether, the lack of binding of PTX3 to human MM6 monocytes may suggest that these monocytes are devoid of PTX3 receptors, such as specific FcγRs, that they do not express sufficiently high levels of PTX3 receptors to allow for detectable amplification of phagocytosis of PTX3 pre-bound bacteria over the background level of phagocytosis, or that PTX3 does not bind to MM6 monocyte surface receptors. This lack of binding between PTX3-coated bacteria and human MM6 monocytes helps explain the lack of amplification of phagocytosis of *Bt* and the lack of observable changes in the bactericidal mechanisms of this cell line against this agent.

## Conclusions

This is the first study to demonstrate widespread immune system gene and pathway regulation and modulation by *Burkholderia* sp. in vitro, on a genome-wide scale, in the context of monocyte infection and in the biologically relevant human host. Among several modulated pathways, the Pattern Recognition Receptor pathway is activated during *Bm* infection of monocytes. Together with upregulation of the long pentraxin-3, a soluble PRR pathway member, we demonstrate lack of interaction between PTX3 and *Bm*. In contrast, PTX3 binds to the related pathogen, *Bp*, and the relatively non-pathogenic species, *Bt*. If lack of binding corresponds to an evasive maneuver by *Bm*, this may stem from the host-adapted nature of this bacterium and its known capacity to circumvent host innate immune responses to persist within the host. Analyses conducted to better define the PTX3-*Burkholderia* sp. relationship did not identify a definitive role for this PRR in the lytic pathway of complement when targeting *Bt* or other bacterial organisms (*Moraxella catarrhalis*). Overall, these data may provide novel insights into the previously suggested role of PTX3 as a modulator of the complement cascade.

Although the role of PTX3 in the opsonophagocytosis and intracellular survival of *Burkholderia* sp. could not be determined in the context of the human MM6 monocytic cell line in this study, future studies are needed to elucidate the potential role of this PRR at the interface between *Burkholderia* sp. and other primary or immortalized cells representing host phagocytes. Specifically, future studies of interest would focus on determining the PTX3-*Burkholderia* sp. relationship in the context of neutrophils, which have been previously described to play a role in cooperative integrative innate immunity with PTX3, against bacterial agents [23, 25]. Specifically, these prior studies have shown PTX3 binding to *Pseudomonas aeruginosa* or uropathogenic *Escherichia coli* to amplify phagocytosis by neutrophils [23, 25].

Overall, given the established roles of PTX3 in microbial recognition and opsonization, complement modulation, opsonophagocytosis, and its demonstrated relevance as an immunotherapeutic towards select pathogens in vivo, a deeper exploration of the potential role of PTX3 in host defense against *Bm* is warranted. In addition to investigating the role of PTX3 in the pathogenesis of *Bm* infection, this genome-wide transcriptome study has identified several modulated genes and pathways that can be further investigated for their potential relevance as immunotherapeutics during *Burkholderia* infection.

## Data Availability

The datasets used and/or analysed during the current study are available from the corresponding author on reasonable request.

## Abbreviations

*Bm*: *Burkholderia mallei*
*Bp*: *Burkholderia pseudomallei*
*Bt*: *Burkholderia thailandensis*
CFU: Colony forming units
CoV: Coefficient of variance
HBSS: Hank’s Balanced Salt Solution
IFN: interferon
iNOS: inducible nitric oxide synthase
IPA: Ingenuity Pathway Analysis
LOOCV: Leave-one-out cross-validation
MM6: Mono Mac-6
NHS: Normal human serum
PE: phycoerythrin
PRR: Pattern Recognition Receptor
PTX3: Pentraxin-3
rhPTX3: recombinant human pentraxin-3
TRAF-6: TNF receptor-associated factor 6
